# Encephalitis and Immune‐Related Complications in a Patient Treated With Nivolumab: A Case Report

**DOI:** 10.1155/carm/4810248

**Published:** 2025-12-29

**Authors:** Georgios Lyras, Prokopios Karydis, Sofia Dionysia Tetradi, Aikaterini Maria Lechouriti, Charalampos Potsios, Dimitra Taprantzi, Christos Michailides, Dimitrios Velissaris

**Affiliations:** ^1^ Department of Internal Medicine, University Hospital of Patras, Patras, Greece, pgnp.gr; ^2^ Medical School of Patras, University of Patras, Patras, Greece, upatras.gr

**Keywords:** complications, encephalitis, immunotherapy, nivolumab, rituximab

## Abstract

**Introduction:**

Immune checkpoint inhibitors (ICIs), such as nivolumab, have made significant advancements in the treatment of several malignancies. However, they have been associated with a new and diverse spectrum of immune‐related adverse effects (irAEs). We report the case of a patient with metastatic renal cell carcinoma who developed encephalitis and other potential irAEs, including hypophysitis with isolated adrenocorticotropic hormone deficiency, transient autoimmune thyroiditis, and autoimmune hemolytic anemia, following combination therapy with nivolumab and cabozantinib. The patient ultimately required rituximab administration.

**Case Presentation:**

A 55‐year‐old woman with a history of metastatic renal cell carcinoma and a recent hospitalization for urinary tract infection was admitted to the Department of Internal Medicine due to confusion, disorientation, and hypotension. A brain MRI revealed multiple new T2/FLAIR hyperintense lesions. After an extensive workup to exclude common neurologic ailments, an adverse reaction due to nivolumab therapy was hypothesized. Treatment was initiated with corticosteroids and IVIG, and the patient showed rapid improvement. Given the fast response to corticosteroids, the diagnosis of ICI‐induced encephalitis was made. Rituximab was then administered, and the patient showed near complete remission of her neurologic symptoms. The patient’s hypotension was attributed to secondary adrenal insufficiency and treated accordingly. Concomitant findings included hyperthyroidism and later‐onset autoimmune hemolytic anemia, both of which were attributed to nivolumab, although the diagnosis could not be confirmed with certainty. Significant improvement was achieved with corticosteroid treatment and rituximab.

**Conclusion:**

irAEs can affect any system, individually or simultaneously, and require high clinical suspicion to diagnose. ICI‐induced encephalitis is potentially life‐threatening but, with prompt treatment, near‐complete recovery is possible. Corticosteroids and IVIG constitute the main treatment, although rituximab may be used successfully in more severe cases.

## 1. Introduction

Immunotherapy has revolutionized oncology and become a mainstay in cancer treatment. Antibodies against programmed Cell Death 1 protein (PD‐1), programmed Death‐Ligand 1 protein (PD‐L1), or cytotoxic T‐lymphocyte‐associated Protein 4 (CTLA‐4), known as immune checkpoint inhibitors (ICIs), have been successfully used in the treatment of non–small‐cell lung cancer, melanoma, renal cell carcinoma (RCC), and other malignancies [1]. Immunotherapy works by suppressing pathological T‐cell deactivation within the tumor microenvironment, thereby priming the immune system to target cancer cells [2].

A major challenge of immunotherapy is the occurrence of immune‐related adverse events (irAEs) as the immune system’s overactivation results in inflammation of healthy tissue. These adverse reactions can affect any organ, with dermatological, gastrointestinal, hepatic, and endocrine toxicities being the most common. Although less common, neurological irAEs (nAEs) involving both the peripheral and the central nervous systems (CNS), including encephalitis, have been reported. Prompt recognition and management of these conditions are essential [3]. Simultaneous presence of multiple irAEs is also possible. Here, we present the case of a woman who, following treatment with the PD‐1 receptor antagonist nivolumab, was diagnosed with encephalitis and ultimately required treatment with rituximab. Concurrently, other possible irAEs manifested, including thyroiditis, hypophysitis, and hemolytic anemia.

## 2. Case Presentation

A 55‐year‐old Caucasian woman was diagnosed in December 2022 with RCC of the left kidney, staged at T1N0M0, for which she underwent surgery. Her past medical history included hypertension and vertigo, for which she was on medication with nifedipine and betahistine, and she was receiving aspirin for primary prevention of atheromatous disease manifestations. In December 2023, she underwent hysterectomy with bilateral salpingo‐oophorectomy due to uterine fibroids. Upon examination of the ovaries, a nodular lesion was identified as a metastatic lesion from the RCC. In April 2024, she initiated combination therapy with nivolumab and cabozantinib. Nivolumab is an antibody against the PD‐1 protein on the surface of T‐cells [2]. Cabozantinib on the other hand is a tyrosine kinase inhibitor (TKI) that targets multiple tyrosine kinase receptors [4].

**Figure 1:**
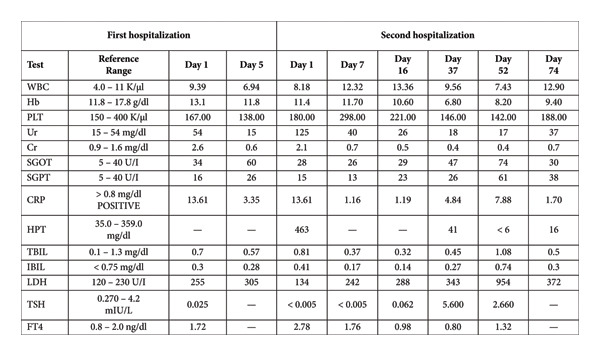
The patient’s laboratory exams during her first and second hospitalizations. CRP and Cr fell to normal values within the first week in both hospitalizations. We can see how anemia worsened about one month after the second admittance to the hospital, which was accompanied by a slight elevation in LDH and a significant fall in HPT, and that it started to resolve later. We also note the changes in TSH after treatment with methimazole. WBC = white blood cells, Hb = hemoglobin, PLT = platelets, Ur = urea, Cr = creatinine, SGOT = serum glutamic oxaloacetic transaminase, SGPT = serum glutamic‐pyruvic transaminase, CRP = C‐reactive protein, HPT = haptoglobin, TBIL = total bilirubin, IBIL = indirect bilirubin, LDH = lactate dehydrogenase, TSH = thyroid‐stimulating hormone, FT4 = free T4.

In August 2024, the patient presented to the emergency department with fever (38.6°C) and hypotension (blood pressure of 70/48 mmHg). She was alert and oriented. Laboratory tests revealed an increase in C‐reactive protein (CRP) (13.61, normal values < 0.5) and acute kidney injury (AKI) (creatinine 2.6 mg/dL and urea 54 mg/dL) (Figure 1). Urinalysis revealed an increased microorganism inoculum and pus cells, while kidney ultrasound revealed no abnormal findings. An electrocardiogram revealed atrial fibrillation of unknown onset. The patient was prescribed broad spectrum empirical antibiotic treatment for suspected urinary tract infection. Following the sepsis protocol of our institution, hydrocortisone 200 mg daily was initiated. Within the first 24 h of her hospitalization, the patient suffered neurological deterioration, gradually developing restlessness, agitation, disorientation, and slurred speech. Magnetic resonance imaging (MRI) of the brain revealed several white matter lesions consistent with microvascular ischemic disease. A lumbar puncture was also performed, and cerebrospinal fluid (CSF) analysis showed 3 cells (2 lymphocytes and 1 monocyte), with glucose and protein levels within the laboratory normal range. CSF, blood, and urine cultures were negative. Over the following days, significant clinical improvement was noted. The patient remained afebrile, AKI was resolved, sinus rhythm was restored and maintained, and she returned to her previous alert and oriented cognitive state. Hydrocortisone was gradually tapered and eventually stopped. Laboratory tests during her hospitalization revealed low thyroid‐stimulating hormone (TSH) levels (0.025 mIU/L) and normal free T4 levels (FT4) (1.72 ng/dL). Following endocrinology expert consultation, treatment was not initiated, and a postdischarge re‐evaluation was proposed. She was discharged 7 days after admission.

Fourteen days later, the patient presented to the emergency department with confusion and disorientation. Upon examination, her vital signs were as follows: blood pressure 88/49 mmHg, pulse rate 122 bpm, 93% oxygenation on room air, and she was afebrile. Initial physical examination did not reveal any concerning findings, and there were no notable differences compared to her previous hospitalization. Laboratory tests again showed an elevated CRP of 13.61 and AKI (creatinine 2.1 mg/dL and urea 125 mg/dL) (Figure 1). Urinalysis was unremarkable. New findings included anemia, as indicated by a hemoglobin level of 11.4 g/dL, with a slightly increased mean corpuscular volume of 99.2. A new brain computed tomography (CT), along with chest and abdominal CT scans, was unremarkable. Given her recent hospitalization, she was once again started on empirical antibiotic therapy with teicoplanin, metronidazole, and fluconazole. Her AKI began to resolve from the first day, CRP levels decreased, and hypotension was managed with intravenous fluids. Antibiotics were continued for a total of 14 days. However, her level of consciousness continued to deteriorate. Adrenal insufficiency was included in the hypotension differential diagnosis. Morning cortisol levels were low (3.28 μg/dL), and adrenocorticotropic hormone (ACTH) levels from the following morning were also low (< 1.5 pg/mL), prompting the initiation of hydrocortisone therapy. Blood and urine cultures were negative, further supporting the diagnosis of secondary adrenal insufficiency as the cause of her hypotension. Further testing revealed elevated follicle‐stimulating hormone and luteinizing hormone levels (35.1 mIU/mL and 17.1 mIU/mL, respectively), while growth hormone levels remained within the normal range (1.49 ng/mL). A repetitive thyroid panel showed exacerbation of hyperthyroidism, with a TSH level of < 0.005 mIU/L, an elevated FT4 of 2.78, and an FT3 of 12.89. Thyroid peroxidase antibodies, thyroglobulin antibodies, and antibodies against the TSH receptor were all negative. This time, treatment with methimazole was initiated (initial total dose: 45 mg daily).

A repetitive lumbar puncture and CSF analysis were performed, once again excluding CNS infection. Glucose levels were within the normal range, protein levels were mildly elevated at 63.1 mg/dL, and the sample was negative for malignant cells. A CSF sample was sent to a specialized laboratory for PCR testing, which returned negative for herpes viruses, other viruses, and common bacteria and Cryptococcus. A new brain MRI revealed multiple new T2/FLAIR hyperintense foci involving the third and fourth ventricles, the posterior and medial parts of the thalamus bilaterally, the mamillary bodies, and the posterior mesencephalon near the colliculi (Figure 2).

Figure 2(a, c) T2/FLAIR images from the brain MRI during the first hospitalization; (b, d) images from the second brain MRI. In (a) and (b), the differences in the thalamuses are more apparent, while in (c) and (d), we can more clearly see the damage to the hypothalamuses and the mamillary bodies.

**Figure:**
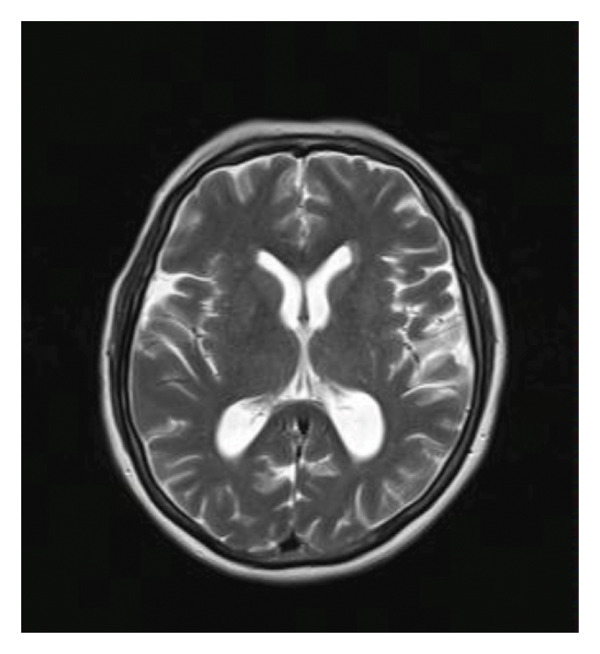
(a)

**Figure:**
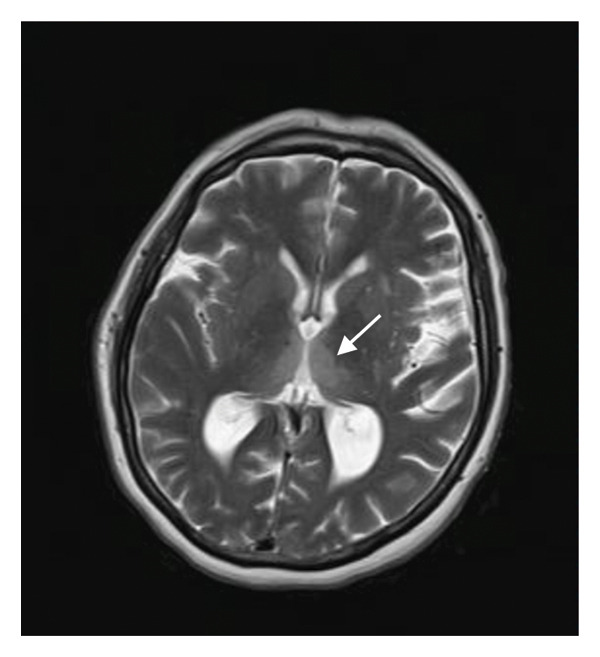
(b)

**Figure:**
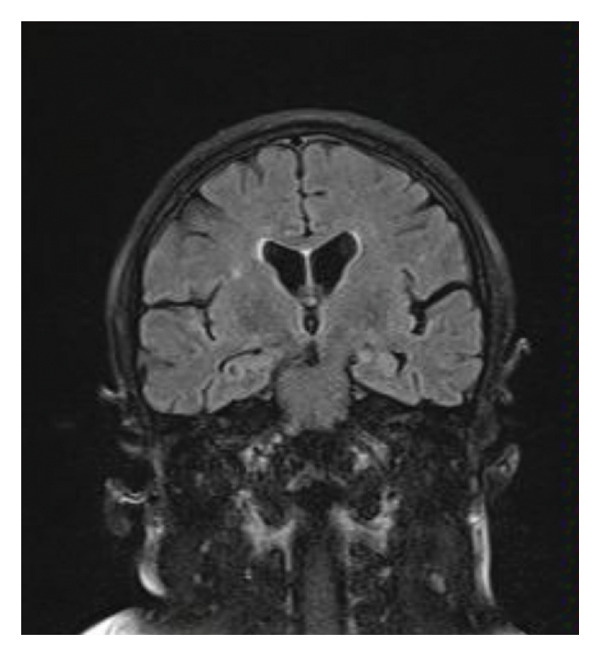
(c)

**Figure:**
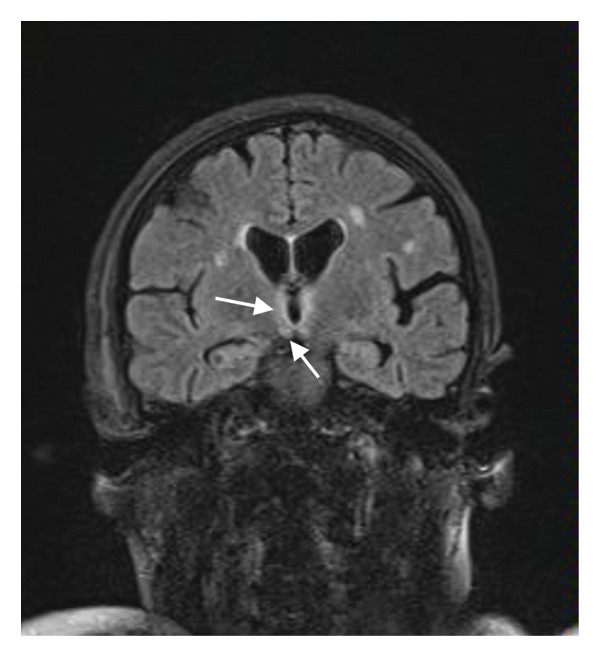
(d)

An electroencephalogram showed a symmetric, nonspecific mixed‐frequency rhythm, without epileptiform activity. Metabolic encephalopathies were considered, and treatment with thiamine and other vitamins was initiated to cover for the possibility of excessive alcohol consumption, without effect. HIV testing was negative. Ceruloplasmin levels were normal, and copper levels in a 24‐h urine sample were in the upper normal limit. She was not receiving any medication that could cause sedation. An echocardiogram and fundoscopy did not show any signs of endocarditis. We then considered autoimmune and paraneoplastic causes and tested specific autoantibodies, all of which were negative. These included the following: GAD, amphiphysin, CV2/CRMP5, Ma2/Ta, Ri/ANNA‐2, Yo/PCA‐1, Hu/ANNA‐1, recoverin, SOX1, Zic4, Tr, GlyR, ANNA‐3, AGNA, CARPVIII, PCA‐2, Homer 3, ITPR1, neurochondrin, RhoGTPase‐activating Protein 26, NMDA‐R, AMPA‐R1/2, GABAB‐R, LGI1, Dopamine‐R2, DPPX, GlurRδ2, IgLON5, mGluR1, mGluR5, CASPR2, AQP4, MOG, and VGKC.

After ruling out every other possible diagnosis, we concluded that an adverse reaction due to nivolumab administration was highly likely. Following established guidelines, and with the assistance of neurology consultation of our institution, hydrocortisone was switched to methylprednisolone at a dose of 1 g daily for 3 days, followed by intravenous immunoglobulin (IVIG) 30 g daily for 5 days. The patient gradually improved starting 2 days after methylprednisolone initiation and even more upon IVIG administration. She was able to follow simple instructions and articulate words into short sentences. This fast response further validated our assumption, and a diagnosis of ICI‐induced encephalitis was made. However, her responses were slow, and she could not execute more complex commands. We decided to step up to rituximab therapy. Three weeks after IVIG infusion, she received 500 mg of rituximab, followed by a second dose 15 days later. Approximately 48 h after the first rituximab infusion, she was fully alert, able to understand and follow all commands, and could speak normally. She did, however, have memory impairment, as she could not remember several details of her past. Also, while she was able to stand, she was not strong enough to walk. This could be a result of the prolonged recumbency, although ICIs are known to cause peripheral neuropathy [1].

Approximately 1 month after initial admission, we noticed a further decline in her hemoglobin values (Figure 1), without any indications of acute hemorrhage. There was an increase in the absolute reticulocyte count, a mild increase in unconjugated bilirubin and LDH levels, and a decreased haptoglobin level. G6PD levels were within the normal range, peripheral blood smear was negative for schistocytes, and direct Coombs testing was positive (1+) for IgG antibodies, under corticosteroid therapy. The patient was diagnosed with hemolytic anemia and, considering her background and the concurrent disorders, we theorized that nivolumab might be the cause. This prompted the continuation of treatment with high doses of methylprednisolone, regardless of the course of encephalopathy. As mentioned above, she also received IVIG and, eventually, rituximab. After consulting with our hematology department and following a steady increase in hematocrit and haptoglobin values, we slowly tapered methylprednisolone. Regarding the endocrinological findings, no new signs or symptoms of Addison disease emerged, and the thyroid panel improved gradually. Methimazole was tapered and eventually discontinued as thyroid hormone levels stabilized within the normal range (Figure 1). Once sufficiently tapered, methylprednisolone was changed to hydrocortisone as replacement therapy. A diagnosis of transient autoimmune thyroiditis and concurrent hypophysitis with isolated ACTH deficiency (IAD), also due to nivolumab, was hypothesized.

Her hospitalization was complicated by a positive blood culture for *Candida auris* early in her stay. Although subsequent blood cultures were negative following central venous catheter removal, skin swab cultures remained positive. Despite her remarkable neurological improvement, given the skin colonization of *C. auris*, she was unable to transfer to a rehabilitation center. As a result, she remained in the hospital, where rehabilitation was provided while awaiting negative skin swab cultures. Unfortunately, her prolonged hospitalization led to multiple hospital‐acquired infections and gradual overall deterioration. Her continuous decline was ultimately complicated by sepsis, leading to her passing 6 months after admission.

## 3. Discussion

Immune checkpoint proteins such as PD‐1 and CTLA‐4 are expressed by T‐cells and play a crucial role in immune regulation. Under normal circumstances, they downregulate T‐cell activity, preventing autoimmune attacks on healthy tissues [5]. However, due to mutations, tumor cells may aberrantly express PD‐L1, which binds to PD‐1, allowing them to avoid immune recognition by T‐cells. To counteract this evasion, several ICIs have been created. Nivolumab is an antibody that binds to PD‐1 and competes with PD‐L1. This results in the reactivation of the immune system against cancer cells [2]. Recently, nivolumab has started being administered in combination with cabozantinib, a TKI that targets multiple tyrosine kinase receptors, for the treatment of RCC. A randomized Phase 3 trial demonstrated the superiority of this combination over sunitinib, leading to its adoption as a treatment option [4].

ICIs come with a major disadvantage in the form of irAEs. Enhanced immune system activation can lead to inflammation of healthy tissues, potentially affecting any organ. The incidence of adverse events varies widely and is as high as 90% depending on the trial. irAEs can appear as early as a couple of weeks after treatment initiation or can have a delayed onset and manifest months or even years after treatment discontinuation. The most commonly affected systems include the skin, gastrointestinal, musculoskeletal, respiratory, and endocrine. However, irAEs involving other systems, such as the central and peripheral nervous and hematopoietic, are well documented. While the majority of irAEs are mild to moderate in severity, severe and life‐threatening reactions can occur [6]. It is also possible for multiple irAEs to coexist in the same patient.

In this report, we presented the case of a patient with ICI‐induced encephalitis. While a rare complication, there have been multiple reports of encephalitis caused by nivolumab. For example, Zafar et al. reported a woman with laryngeal squamous cell carcinoma, who was treated with nivolumab, developed mental deterioration and obtundation, and was ultimately treated with corticosteroids and IVIG [7]. In our case, the need for therapy with rituximab and the multiple concurrent autoimmune complications set the case apart from previous reports.

Neurological AEs occur with an incidence of up to 6.1% for anti‐PD1 antibodies. Most cases are of mild to moderate severity (Grade 1 or 2), while higher‐grade toxicities have an incidence of less than 1%. The time delay from initiation of therapy to the clinical manifestation of nAEs varies greatly. Cuzzubo et al. reported a median onset time of 6 weeks, but with a broad range from 1 to 74 weeks [8]. Encephalitis caused by ICIs is associated with significant morbidity and mortality, particularly if diagnosis is delayed. Thus, prompt initiation of treatment is essential. Most patients require prolonged hospitalization and further rehabilitation, but near‐complete recovery is possible with timely intervention [9]. First‐line treatment consists of corticosteroids, with an initial trial of 1‐2 mg/kg of methylprednisolone per day. In case of progressing disease or very severe initial symptoms, pulse‐dose methylprednisolone is given for a total of 1 g iv daily and/or IVIG 2 g/kg over 5 days. If the patient does not respond within 2‐3 days, the therapy should be escalated with plasmapheresis or rituximab. ICIs should be withheld in all patients [1].

Our patient was initially admitted to the hospital with clinical manifestations consistent with sepsis. She deteriorated neurologically, but CNS imaging and CSF analysis were normal. She showed improvement with empirical sepsis treatment and was subsequently discharged. She was later readmitted due to neurological manifestations. It is likely that the short course of corticosteroids administered as part of the sepsis protocol was responsible for the initial recession of symptoms. However, it is impossible to say with certainty whether this was ultimately beneficial or deleterious. On one hand, early administration of corticosteroids may have prevented further disease progression. On the other hand, the transient improvement and hospital discharge may have delayed the definitive diagnosis.

Nivolumab and cabozantinib have distinct mechanisms of action, leading to vastly different adverse effect profiles. This led to the hypothesis that the patient’s symptoms were primarily due to nivolumab, as her clinical findings were consistent with its known complications [10]. Cabozantinib may cause posterior reversible encephalopathy syndrome (PRES) due to inadequate hypertension control during therapy [11]. However, our patient neither presented with hypertension upon admission nor exhibited MRI findings typical of PRES.

Our patient showed considerable improvement with corticosteroids and IVIG therapy; however, rituximab was necessary to achieve near‐complete recovery. Rituximab is a chimeric monoclonal antibody that acts against the protein CD20 on the surface of B‐cells. It leads to B‐cell depletion via a multitude of ways, including antibody‐dependent cellular cytotoxicity, complement‐dependent cytotoxicity, opsonization, and directly induced apoptosis. This effect on B‐cells makes rituximab a compelling therapy for diseases where their pathophysiology revolves around antibodies [12]. Although rituximab is mentioned in treatment guidelines, there are very few reports documenting its use in this context. Williams et al. reported a case where rituximab was administered, leading to gradual improvement, though the timeframe for its effects was not specified [13]. Lyons et al. present a case in which rituximab was used following multiple symptom relapses, and the patient improved only modestly [14]. In our case, the patient improved significantly within 2‐3 days of rituximab initiation. It is clear that many questions regarding its use and efficacy remain unanswered. These include the time interval between treatment initiation and expected recovery, the disease severity threshold for initiating rituximab therapy, its effectiveness in managing symptom relapse, the relevance of specific autoantibody presence in selecting rituximab therapy, and its potential impact on cancer progression and prognosis.

In addition to encephalitis, our patient experienced multiple concurrent disorders, including hyperthyroidism and secondary adrenal insufficiency. ICI‐associated hyperthyroidism occurs with a frequency of 2.9% [15], and a median onset time ranging from 21 to 47 days after first treatment administration, depending on the regimen used [16]. Thyroid dysfunctions rarely become life‐threatening [15]. Thyrotoxicity typically does not need to be treated, as it is transient and can be managed symptomatically with *β*‐blockers, hydration, and supportive care [1, 16]. Thionamide should be considered in cases of persistent hyperthyroidism, severe symptoms, or concern for thyroid storm. If symptoms become severe, corticosteroids should be used. Nivolumab administration can usually be continued [1]. On the other hand, ICI‐induced hypophysitis has been identified as a rare yet potentially life‐threatening adverse effect, with a prevalence of less than 1% after anti‐PD1 therapy [17]. IAD is one of the rarer complications, with a prevalence of < 1%, and leads to secondary adrenal insufficiency. Typically, IAD manifests within the first few months after the initiation of ICI therapy, although it may also present several months after the discontinuation of treatment [18]. Treatment follows the same protocols as with other cases of secondary adrenal insufficiency. For Grade 3 or 4 toxicities, pulse prednisone is administered at a dose of 1‐2 mg/kg daily, tapered over a couple of weeks. It is recommended that ICIs be withheld until the patient stabilizes on replacement hormone therapy [1].

Given our patient’s medical history, the timeline in which these disorders developed, and after excluding other common causes, we hypothesized that nivolumab was responsible for both hyperthyroidism and hypophysitis, as multiple irAEs can occur simultaneously in the same patient [19]. However, due to skin colonization with *C. auris*, and the associated risk of hospital‐wide transmission, several diagnostic tests, including thyroid ultrasound and scintigraphy, and pituitary MRI had to be canceled. Consequently, we were unable to confirm the diagnoses with certainty.

During her hospitalization, our patient was also diagnosed with autoimmune hemolytic anemia (AIHA). ICI‐induced AIHA has an incidence of < 0.5%, and up to 60% of cases are attributed to nivolumab and are usually of high severity. The median time of onset from treatment initiation is approximately 10 weeks, with a wide range of 2–78 weeks. Diagnosis is based on the same principles as other cases of hemolytic anemia, and the direct antiglobulin (DAT) test, also known as Coombs test, is essential for identifying immune‐mediated etiologies [20]. Treatment primarily involves corticosteroid administration. For Grade 3 or higher toxicities, prednisone 1‐2 mg/kg is administered. Permanent ICI discontinuation is also advised. In case of refractory or worsening disease, second‐line therapies should be considered. These include IVIG, rituximab, Cyclosporin A and mycophenolate mofetil [21].

In our patient, DAT results were deemed inconclusive due to the patient already receiving high‐dose corticosteroids for encephalitis. Furthermore, DAT‐negative AIHA has been well observed and documented in the literature [22]. It should be noted that other factors, such as inflammation‐induced bone marrow suppression, may have contributed to the anemia developed in our patient. Also, while AIHA usually manifests within the first 3 months following ICI administration, more delayed cases have been reported [20]. Nevertheless, based on our patient’s complete laboratory profile and the absence of definitive evidence pointing to other causes of anemia, we can partially attribute the acute event to nivolumab.

Our patient was treated for encephalitis with IVIG and later with rituximab. However, this treatment course could have also been considered as a possible intervention for the anemia, since AIHA manifested while the patient was already undergoing corticosteroid therapy. Rituximab has been successfully used in patients that did not respond to corticosteroids. For example, Khan et al. reported a case of a 43‐year‐old woman diagnosed with AIHA after treatment with ipilimumab and nivolumab. She was initially treated with corticosteroids, but after ICIs reintroduction and symptom relapse, rituximab was used, leading to remission [23].

This case also serves as a reminder of the risks of prolonged recumbency and the critical importance of rehabilitation. While our patient showed significant neurological, endocrinological, and hematological improvement, excessive muscle weakness prevented her from getting out of bed. After starting physiotherapy, during hospitalization, she managed to stand but could not walk. She could not be transferred to a rehabilitation center while still a carrier of *C. auris*. The insufficient rehabilitation contributed to a series of infections and other complications, ultimately leading to her death.

It should be mentioned that the use of rituximab in our patient, as well as the course of corticosteroids and IVIG, certainly raised the risk and worsened the prognosis of hospital‐acquired infections. In a meta‐analysis by Nepal et al. for the efficacy and safety of rituximab in patients with autoimmune encephalitis, the rate of infection was 6%, which matches previous studies that report a rate of about 10% [24]. Thus, we should not forget that rituximab, while a promising answer to ICI‐induced encephalitis, raises the risk of life‐threatening infections and other complications of immunosuppression.

## Ethics Statement

Consensus form was given by the patient before death. Our study is in line with the International Committee of Medical Journal Editors Guidelines, CARE Guidelines, the Helsinki Declaration, and Best Practice Guidelines according to the Committee on Publication Ethics (COPE).

## Disclosure

All authors gave consent for publication.

## Conflicts of Interest

The authors declare no conflicts of interest.

## Author Contributions

Conceptualization was done by Georgios Lyras, Prokopios Karydis, Christos Michailides, and Dimitrios Velissaris. Data curation and writing were done by Georgios Lyras, Prokopios Karydis, Sofia Dionysia Tetradi, Aikaterini Maria Lechouriti, Charalampos Potsios, and Dimitra Taprantzi.^.^ Supervision was done by Christos Michailides and Dimitrios Velissaris.

## Funding

This study was not supported by any sponsor or funder.

## Data Availability

The data that support the findings of this study are available from the corresponding author upon reasonable request.
